# The effect of a supportive home care program on caregiver burden with stroke patients in Iran: an experimental study

**DOI:** 10.1186/s12913-021-06340-4

**Published:** 2021-04-15

**Authors:** Mansoureh Ashghali Farahani, Tahereh Najafi Ghezeljeh, Shima Haghani, Farshid Alazmani-Noodeh

**Affiliations:** 1 Nursing Care Research Center, School of Nursing and Midwifery, Iran University of Medical Sciences, Tehran, Iran; 2 Nursing Care Research Center, Iran University of Medical Sciences, Tehran, Iran

**Keywords:** Stroke, Caregivers, Homecare services, Nursing

## Abstract

**Background:**

Stroke can impose a heavy burden on caregivers. Caring for stroke patients at home is more challenging than in hospitals with facilities. The purpose of this study was to evaluate the effect of a supportive home care program on caregiver burden with stroke patients.

**Methods:**

This was an experimental study. One hundred sixteen caregivers of stroke patients were recruited using convenience sampling from two university-affiliated hospitals in Tehran from June 2019 to February 2020. They were randomly allocated into two groups (supportive home care program and routine hospital education program) using a randomized block design. The supportive home care program included eight educational sessions delivered in the hospital before discharge, and with home visits after hospital discharge. Caregiver burden was measured using Caregiver Burden Inventory. The data were analyzed using independent samples t-test and Analysis of Covariance.

**Results:**

Caregiver burden in the routine education group increased significantly after 2 weeks, from 52.27 ± 23.95 to 62.63 ± 22.68. The mean of caregiver burden scores in the supportive home care program decreased from 44.75 ± 17.21 to 40.46 ± 17.28. The difference between the scores of the two groups before the intervention was not significantly different (t = 1.941, df = 114, *p* = 0.055). There was a significant difference between the two groups regarding caregiver burden scores after the intervention period (η^2^ = 0.305, *P* < 0.001).

**Conclusions:**

Caregiver burden increased significantly after the discharge without proper interventions in the caregivers of stroke patients. Providing support for home care providers can help to decrease or prevent the intensification of caregiver burden.

## Background

Caregiver Burden (CB) is the negative effect of caregiving tasks that caregivers perceive [1], in terms of their emotional state, physical health, social life, and financial status being affected by caring for their ill relative [2]. Caregiver burden is defined as the all-encompassing challenges felt by caregivers regarding their physical and emotional well-being, family relations, and their work and financial status [3]. Caregiver burden is associated with negative outcomes for both caregivers and patients, including the reduction of their general health and quality of life [4, 5], and increasing the risk for patient’s morbidities [6–8], which is a multidimensional response to perceived stress and negative assessments that derive from providing care to a patient [9]. Caregivers of stroke patients experience a high level of caregiver burden because they need to provide care for long hours [10, 11], and the patients usually dependent on them for their activities of daily life (ADL) [12].

Sensory and motor disabilities are major complications of a stroke that cause various degrees of dependence in patients [13]. These complications may make it difficult or even impossible for a person to perform daily activities of life. According to the World Health Organization, 15 million people suffer stroke worldwide each year. Of these, 5 million die and another 5 million are permanently disabled. The incidence of stroke is about 43 patients per 100,000 population [14]. In a population-based study conducted in Mashhad, Iran, Ischemic stroke (IS) was 81.9% and Hemorrhagic stroke (HS) was 15.1% of all the patients [15]. While only 24% of patients become independent in their daily activities after rehabilitation, the rest depends on the help of a caregiver for their daily activities [16]. After the acute phase, patients are usually discharged from the hospital, and the care will continue by family members [17]. Taking care of a patient with a stroke causes great physical and mental stress for the caregiver, as well as the family [18]. More than half of patients need permanent or temporary assistance from the people they live with for their ADL [19].

In developing countries like Iran, there are very few public support and care centers that provide post-discharge education and care in stroke patients [10, 20]. Moreover, the cost of care in private centers and home care services is high. This makes care by family caregivers very common, and most families take the responsibility of caring for their patients [11, 21]. A stroke is an unexpected event, and stroke patients need long-term support at home to recover from stroke-related disabilities and multiple complications [22]. Family members usually play the role of caregiver very suddenly, and they are unprepared for it [23, 24]. In this case, family members became the main caregivers and they experience high levels of stress and anxiety, which causes many problems in the implementation of the patient support program [25, 26]. These problems usually occur in the post-discharge period.

The number of interventional studies in the field of CB reduction in caregivers of stroke patients is limited. A study investigated the effect of social problem-solving telephone partnerships on primary family caregiver burden after stroke survivors are discharged home from a rehabilitation facility [27]. Their results showed that family caregivers who participated in the social problem-solving telephone partnership intervention had better problem-solving skills, less depression, greater caregiver preparedness, and significant improvement in measures of vitality, social functioning, and mental health. However, Caregiver burden was not significantly different among study groups. The results of another study showed that a multidimensional rehabilitation intervention can reduce Caregiver burden of caregivers of stroke patients. Most studies have focused on the factors affecting the caregiver burden, and few interventional studies have been performed in this area [28].

The family needs a proper understanding of the disease and comprehensive support during the care of a patient after discharge. The nurse must provide proper information and support for the family in addition to caring for the patient [29, 30]. Supportive home care is defined as the provision of non-medical care or custodial care to individuals in a home setting. Supportive home care is a method of CB reduction. Nurses are in a unique position to interact with family caregivers [17]. They can provide the knowledge, skills, and support needed to maintain the quality of care at home.

In many developing countries, including Iran, the family provides care after the discharge of stroke patients [10, 11]. Although there are facilities that provide full or part-time care after the discharge of hospitals for stroke patients, many families tend to take care of their patients at home or are unable to afford to use the services of institutions. However, the results of previous studies have shown that the burden of care for stroke patients is very high, which can endanger the health of the patient and the caregiver [4–8]. This study has been tried to evaluate the effect of a supportive home care program on Caregiver burden of caregivers of stroke patients.

## Methods

This experimental study was a part of mixed-method research. One hundred sixteen caregivers were randomly assigned to two groups. The primary outcome was caregiver burden, which was assessed by Caregiver Burden Inventory.

**Fig. 1:**
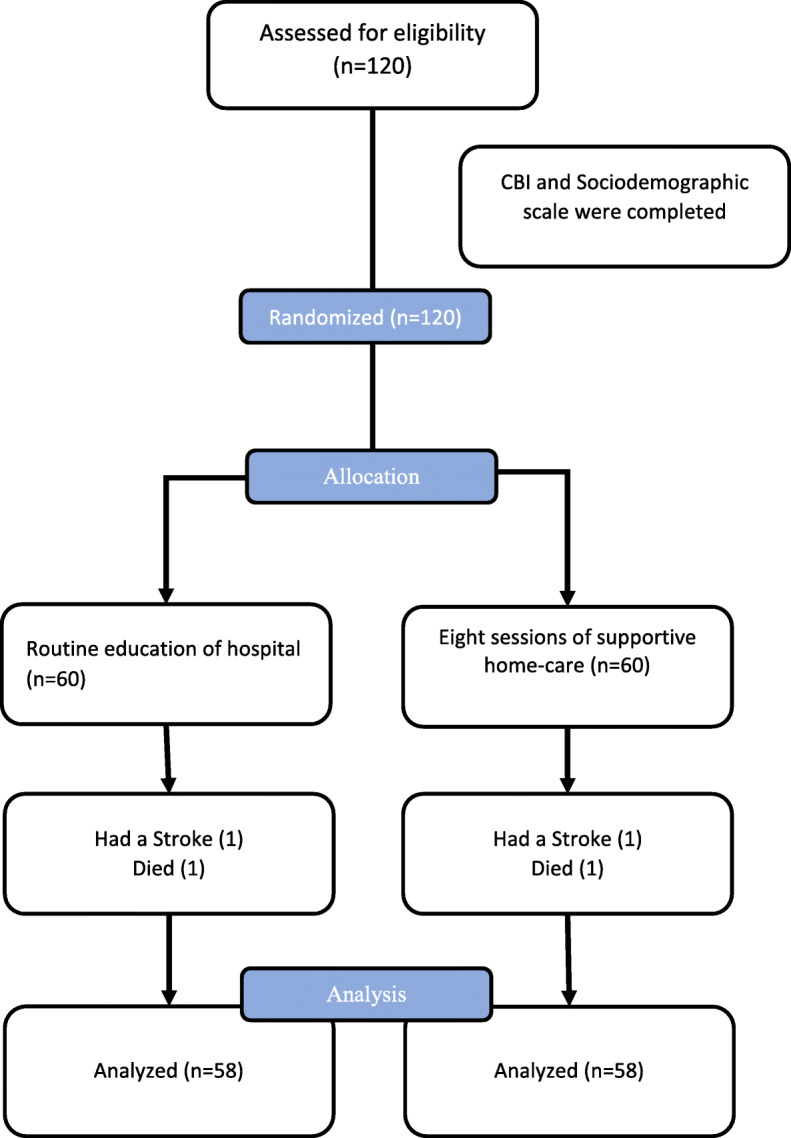
subject’s enrollment, randomization, and data gathering process in both groups

The convenience sampling method was used to enroll the study subjects. All patients who were admitted to the hospital during the study period were approached by the first author. The caregivers of patients were assessed for eligibility. If they had inclusion criteria, they were randomly assigned to one of the two groups, with a randomized block design. The inclusion criteria were [1] being an immediate family member, [2] being literate (able to read and write Persian), [3] Being able to effectively communicate, [4] having no previous attended training sessions related to this intervention, [5] Based on the Barthel index; the patient had a moderate to severe dependency level, [6] it was their first experience of caring for a patient with CVA, [7] do not suffer from a known physical or mental illness and [8] not being medical personnel. The corresponding author and the statistician were not present in the subject enrollment. The first and second authors were not involved in data analysis. The subject recruitment was conducted from Rasool-Akram hospital and Firouzgar hospital, two Iran University of Medical Sciences Affiliated hospitals in Tehran, Iran, from June 2019 to February 2020. The sample recruitment was continued to the achievement of the minimum sample size. Recruitment and allocation to study groups are presented in Fig. 1.

The minimum sample size was determined using the following equation $$ \mathrm{n}=\frac{{\left({\mathrm{z}}_{1-\raisebox{1ex}{$\upalpha $}\!\left/ \!\raisebox{-1ex}{$2$}\right.}+{\mathrm{z}}_{1-\upbeta}\right)}^2\times \left({\upsigma}_1^2+{\upsigma}_2^2\right)}{{\mathrm{d}}^2}=60 $$ and parameters, including alpha = 0.05, power = 0.80, and two standard deviations as much as (Chen et al., 2015) 15.1 and 11.7 to discover a minimum difference of 7. The study subjects were the main caregivers of patients with a confirmed diagnosis of CVA.

Barthel Index was used for Activities of Daily Living to assess the mobility function and self-care activities such as eating, bathing, and dressing [31]. This Index has 20 items, which is scored based on a five-point Likert. The maximum score of Barthel’s index is 20, and the minimum is 0. The higher scores indicate a higher level of dependency. Based on the scoring, the score of 20 is interpreted as independent and the score of 0–19 as dependent. The dependency level is divided into the categories, including mild dependent [12–19], moderate dependent [9–11], severe dependent [5–8], and dependent (0–4). It should be said that the Barthel Index is not under license. The exclusion criteria were the death of the patient during the intervention and attending only one training session.

The first author explained the purpose of the study and procedures to caregivers with a full description of the study intervention and routine care. Then, written informed consent was obtained from them. The block randomization was performed using a computer-generated permuted block randomization scheme (blocks of four). Subjects were randomly assigned to supportive home care programs and routine hospital education program groups by the statistician who was not involved in determining the eligibility of the subjects and had no information from the persons included in the experiment and no influence on the assignment sequence.

Eligible subjects were chosen from patients with Stroke in the ward who was stable and ready to discharge. Barthel Index of Activities of Daily Living (BI) was completed for each eligible patient. After the allocation of eligible subjects, they completed the socio-demographic questionnaire and CBI (Pre-test). Caregivers in the routine hospital education program group received routine educations about CVA and caring for patients with CVA, which was programmed and performed in the hospitals before this study. They completed the study questionnaires 2 weeks after discharge. Subjects in the intervention group received an eight-session intervention program. The first two sessions of the program in the intervention group were planned and conducted at the hospital after the stabilization of the patient’s condition. The remaining six sessions of the program were performed at the patient’s home in 2 weeks. They completed post-tests after the intervention. Each session lasts between 45 to 60 min, and the intervention was finalized 2 weeks after discharge from the hospital. The Content of the sessions is presented in Table 1. The intervention was conducted by the first author who was a Ph.D. candidate of nursing at the time of the study with 10 years of work experience as a nurse. Furthermore, he was worked with stroke patients and attended several workshops and courses regarding homecare of stroke patients before the intervention. The content of the intervention was approved by a team, including two nursing professors, two neurologists, and one physical therapist.

**Table 1:** Content of intervention sessions

Session	Content
One	The purpose of the intervention and the importance of collaborating of caregivers along with the details of the support plan and the educational booklet
Two	The mechanism of stroke, the etiology, and the signs and symptoms of CVA, the types of treatments, the importance of patient care, patient transfer, the problem-solving and coping skills, how to communicate effectively with the patient, and active listening
Three	Anger management, rational dealing with anger and appropriate techniques to control it, Diet, monitoring the nutritional status, how to feed with a nasogastric tube if needed, and weight control.
Four	Control of the patient’s blood pressure, physiotherapy of the limbs and respiratory system, prevention of limb deformity, change position, and prevention of pressure ulcer
Five	Deep breathing exercises and relaxation, bathing, general hygiene, dressing, oral and teeth hygiene, and suction of the patient
Six	Medications and their side effects and using a glucometer if needed.
Seven	Speech therapy and assessment of the need for counseling with a psychologist or psychiatrist
Eight	Follow-up therapies, assess the need for further referrals, paraclinical procedures, and answering the questions

The main outcome of the study was the caregiver burden, which was measured by Caregiver Burden Inventory (CBI). This 24-item is scaled with Likert-format scoring from 0 to 4 with five dimensions, including time dependence [1–5], developmental [6–10], physical [11–14], social [15–19], and emotional burden [20–24]. The scale is developed by Novak and Guest [32]. The Persian version of this scale is adopted from Abbasi et al. (2011). They reported the internal consistency of the CBI (α = 0.9). All study subjects completed the CBI after enrollment and after 2 weeks [33]. The data collection was conducted by the corresponding author. She is a nursing professor and the supervisor of the dissertation that this study was a part of it.

The data were entered into SPSS 16. The normality of the distribution of CBI scores was tested using the Kolmogorov-Smirnov test. The CBI score was reported as Mean ± Standard Deviation (SD). The difference in the scores of CBI and its domains between the two groups was tested by the independent samples t-test. Analysis of Covariance (ANCOVA) was used to compare the scores of CBI and its domains between the two groups after the intervention period. CBI and its domain scores before the intervention were used as a covariate in the ANCOVA. Linear regression was used to assess the effect of socio-demographic characteristics on the results.

### Ethics considerations

The study protocol was approved by the Ethics Committee of the Iran University of Medical Sciences (IR.). All study subjects completed the informed written consent. All study subjects could withdraw from the study whenever they desired. The information on all research units was confidential.

## Results

One hundred sixteen caregivers were enrolled in the study and finished the post-test. The mean ± SD of the age of study subjects was 43.98 ± 13.80 years and 43.41 ± 11.25 years in the supportive home care program and routine hospital education program groups, respectively (t = 1.631, df = 114 *p* = 0.106). The mean ± SD of the age of patients was 68.50 ± 13.16 years in the supportive home care program group and 64.65 ± 12.20 in the routine hospital education program group (t = 1.631, df = 114 *p* = 0.106). The Sociodemographic characteristics of the study subjects are presented in Table 2, and the Sociodemographic characteristics of patients are presented in Table 3. Both groups were similar in terms of socio-demographic factors. Based on linear regression, no confounding factor was found in the study and there was no bias in sampling and analysis.

**Table 2:** Frequency Distribution of Demographic Characteristics of the caregivers in each group

		Intervention(*n* = 58)	Control(*n* = 58)	*p
Age (Years); M (SD)		43.98 (13.8)	43.41 (11.25)	†0.808
Gender	Male	20 (34.5)	13 (22.4)	††0.150
	Female	38 (65.5)	45 (77.6)	
Relationship	Offspring	41 (70.7)	32 (55.2)	††0.211
	Spouse	12 (20.7)	17 (29.3)	
	Other	5 (8.6)	9 (15.5)	
Marital Status	Single	16 (27.6)	12 (20.7)	^&^0.307
	Married	37 (63.8)	44 (75.9)	
	Divorced or widowed	5 (8.6)	2 (3.4)	
Education	Elementary school	2 (3.4)	6 (10.3)	^&^0.538
	Below Diploma	7 (12.1)	7 (12.1)	
	Diploma	23 (39.7)	21 (36.2)	
	Academic	26 (44.8)	24 (41.4)	
Job	Government Employee	16 (27.6)	24 (41.4)	††0.295
	Self-employed	14 (24.1)	12 (20.7)	
	Retired	9 (15.5)	4 (6.9)	
	Housewife	19 (32.8)	18 (31)	
Living with Patient	Yes	36 (62.1)	28 (48.3)	††0.135
	No	22 (37.9)	30 (51.7)	
Chronic Diseases	Yes	16 (27.6)	19 (32.8)	††0.544
	No	42 (72.4)	39 (67.2)	
Duration of patient care (Month), M (SD)		13.19 (8.78)	15.02 (10.29)	†0.306

*Significance level: *P* < 0.05 † Independent sample t-test ††Pearson’s chi-square test ^&^ Fisher Exact Test

**Table 3:** Frequency Distribution of Demographic Characteristics of the Patients in each group

		Intervention(*n* = 58)	Control(*n* = 58)	*p
Age (Years); M (SD)		68.5 (13.16)	64.65 (12.2)	†0.106
Gender	Male	32 (55.2)	31 (53.4)	††0.852
	Female	26 (44.8)	27 (46.6)	
Marital Status	Single	1 (1.7)	4 (6.9)	^‡^0.121
	Married	45 (77.6)	35 (60.3)	
	Divorced or widowed	12 (20.7)	19 (32.8)	
Education	Elementary school	24 (41.4)	21 (36.2)	^††^0.243
	Below Diploma	10 (17.2)	11 (19)	
	Diploma	11 (19)	19 (32.8)	
	Academic	13 (22.4)	7 (12.1)	
Job	Government Employee	9 (15.5)	3 (5.2)	††0.172
	Self-employed	7 (12.1)	13 (22.4)	
	Retired	19 (32.8)	21 (36.2)	
	Housewife	23 (39.7)	21 (36.2)	
City	Tehran	58 (100)	55 (94.8)	‡0.243
	Other cities	0 (0)	3 (5.2)	
Insurance	Yes	56 (96.6)	57 (98.3)	‡0.999
	No	2 (3.4)	1 (1.7)	
whom does the patient live with	alone	3 (5.2)	4 (6.9)	‡0.139
	Spouse and Offspring	34 (58.6)	44 (75.9)	
	Offspring	15 (25.9)	9 (15.5)	
	parents	3 (5.2)	1 (1.7)	
	Other	3 (5.2)	0 (0)	
Duration of diagnosis (Month), M (SD)		14.29 (8.89)	15.02 (10.29)	†0.686
smoking	Yes	12 (20.7)	13 (22.4)	††0.821
	No	46 (79.3)	45 (77.6)	

*Significance level: *P* < 0.05 † Independent sample t-test ††Pearson’s chi-square test ‡ Fisher Exact Test

Caregiver Burden and its domain scores are presented and compared in Table 4. Caregiver Burden scores of study subjects in the control group increased significantly during the intervention period (*p* < 0.05). The scores of CBI and all its domains increased significantly in the control group (*p* < 0.05). It means that the caregivers felt a higher level of CB. Caregiver Burden scores in the intervention group decreased significantly. It means that caregivers felt a lower level of CB. In the intervention group, the mean scores of time-dependences (*p* < 0.05), physical (*p* > 0.05), and emotional (*p* < 0.05) domains were decreased, and the mean scores of developmental (*p* > 0.05) and social domains (*p* > 0.05) were increased. It means that the intervention was more successful in the prevention of physical and emotional burdens along with time dependence activities.

**Table 4:** **Comparison of Caregiver burden scores and its domains between two groups**

Group	Time	Intervention(*n* = 58) M (SD)	Control(*n* = 58) M (SD)	*p
Domain				
Time-Dependence	Before	16.82 (3.01)	15.98 (4.50)	†*p* = 0.237
	After	14.32 (3.50)	17.36 (3.92)	^**‡**^***P*** **< 0.001** η^2^ =0.345
	††*p*	***p*** **< 0.001**	***p*** **< 0.001**	
	Difference	−2.5 (3.33)	1.37 (1.97)	†***p*** **< 0.001**
Developmental	Before	10.08 (5.33)	11.91 (6.38)	†*p* = 0.097
	After	10.32 (4.75)	14.22 (5.44)	^**‡**^***P*** **< 0.001** η^2^ = 0.133
	††*p*	*p* = 0.702	***p*** **< 0.001**	
	Difference	0.24 (4.78)	2.31 (3.22)	†***p*** **= 0.007**
Physical	Before	5.75 (5.08)	8.84 (5.42)	†*p* = 0.002
	After	5.39 (4.81)	9.94 (5.16)	^**‡**^***P*** **< 0.001** η^2^ = 0.103
	††*p*	*p* = 0.532	***P*** **= 0.02**	
	Difference	−0.36 (4.38)	1.1 (3.5)	†***p*** **= 0.049**
Social	Before	4.53 (5.13)	6.15 (5.21)	†*p* = 0.097
	After	5.06 (5.31)	9.74 (5.95)	^**‡**^***P*** **< 0.001** η^2^ = 0.147
	††*p*	*p* = 0.353	***p*** **< 0.001**	
	Difference	0.53 (4.34)	3.58 (4.49)	†***p*** **< 0.001**
Emotional	Before	7.55 (5.31)	9.37 (6.37)	†*p* = 0.096
	After	5.34 (4.26)	11.25 (5.73)	^‡^*P* = 0.147 η^2^ = 0.288
	††*p*	***p*** **= 0.001**	***p*** **< 0.001**	
	Difference	−2.2 (4.78)	1.87 (4.44)	†***p*** **< 0.001**
Caregiver Burden	Before	44.75 (17.21)	52.27 (23.95)	†*p* = 0.055
	After	40.46 (17.28)	62.63 (22.68)	^**‡**^***P*** **< 0.001** η^2^ = 0.305
	††*p*	***p*** **= 0.036**	***p*** **< 0.001**	
	Difference	−4.29 (15.21)	10.36 (10.83)	†***p*** **< 0.001**

*Significance level: *P* < 0.05 † Independent sample t-test †† Paired T-test ‡ ANCOVA test with adjusting the baseline score

η^2^= partial eta-squared = Effect sizes: 0.01 = small; 0.06 = moderate; 0.14 = large.

There was no significant difference between the two groups in terms of time dependence domain before the intervention period (*p* > 0.05). The difference became significant after the intervention period and the control group had a higher score of time dependence caregiver burden (*P* < 0.001) Time dependence domain mean score in the intervention group was decreased while it was increased in the control group. The difference in the developmental domain between the two groups was not significant before the intervention period (*p* > 0.05). The control group had a significantly higher score of developmental domain score after the intervention period (*P* < 0.001). The developmental score increased in both groups but the increase in the control group was significantly higher (*P* < 0.001) There was a significant difference between the two groups in terms of the physical domain before the intervention period and the intervention group had a higher score (*p* < 0.05). The physical domain score decreased in the control group and increased in the control group and the difference remained significant with opposite direction, where the control group received higher scores (*p* < 0.05). There was no significant difference between the two groups in terms of the social domain before the intervention period (*p* > 0.05). The difference became significant after the intervention period and the control group had a higher score of social caregiver burden (*P* < 0.001) Social domain mean score in both groups was increased. The difference in the emotional domain between the two groups was not significant before the intervention period (*p* > 0.05). ANCOVA showed that the significant difference between two groups remained after the intervention period (*p* > 0.05). There was no significant difference between the two groups in terms of CB score before the intervention period (*p* > 0.05). The difference became significant after the intervention period and the control group had a higher score of caregiver burden (*P* < 0.001). CB mean score in the intervention group was decreased while it was increased in the control group (*P* < 0.001).

## Discussion

The results showed that the program is effective in reducing caregiver burden and its domains. The CB increased in the routine hospital education program and decreased in the supportive home care program.

The level of care burden in caregivers of stroke patients is very high. The research has shown that their caregiver burden is very frustrating, especially in the first few weeks. This is a factor that can have very negative effects on patients’ outcomes. Insufficient knowledge about the nature of the disease and its severe effects can lead to severe frustration in caregivers. Failure to follow up on primary care, such as providing general hygiene and physiotherapy can lead to serious complications, such as infection and deformity of the limbs [34, 35]. These effects can jeopardize a patient’s future performance or even threaten his/her life. The results showed that proper education and follow-up along with providing proper support can reduce the CB. However, the burden of care and all its dimensions in the control group increased over time. Previous research has shown that CB in caregivers of stroke patients will increase over time if the proper intervention is not provided [36].

Caregiver Burden is a multidimensional phenomenon whose dimensions are time-dependence, developmental, physical, social, and emotional burden. The results showed that the intervention significantly affects the score of the time-dependence domain. Time-dependence is the perceived burden attributable to restrictions on a caregiver’s time forced by the demands of caring for the patient [37]. A patient with a stroke needs full-time care due to complete dependence [18]. These time constraints had a significant effect on the time-dependent caregiver burden. Supportive home care can help caregivers to learn problem-solving and time management skills. Previous studies have shown that supportive home care can reduce time-dependent CB.

While the change in the developmental burden score in the control group was significantly positive, it was negative in the intervention group. The developmental burden is the perceived feelings that caregivers have. They are “out of sync” with their peers or feelings of missing out on life [37]. The decrease in the scores of this domain was not significant, but the intervention was effective in preventing it from increasing over time. Learning to do basic care at home and providing time to be with others can help to reduce the developmental burden [16].

The results showed that the increase of physical burden, which describes chronic fatigue and damage to the physical health of caregivers is significant in the control group. The physical burden also increased in the intervention group, but the increase was not significant. Primary skills training was effective in this outcome [37]. Although the burden of physical care increased in both groups, the increase is much greater in the control group. Simple care, such as measuring blood pressure or blood sugar can significantly reduce the physical burden of care. The results of previous studies also showed that the physical burden of care can be reduced using supportive home care programs [17]. The results did not show negative changes in the physical burden scores, but the intervention was successful in the prevention of a dramatic increase.

A supportive homecare program is also effective in the prevention of an increase in the social burden of care. Social burden refers to conflicts with other family members about care decisions, or feelings of isolation such as not having time to maintain social relationships [37]. The education about problem-solving and conflict management in this supportive program may help in this result. The results also showed that the supportive program is effective in the reduction of emotional burden, which significantly increases in the control group over the time of care. Emotional burden describes negative feelings toward the care receiver, compounded by the caregiver’s subsequent feelings of guilt for having these socially unacceptable feelings [37].

Our study evalutes the effect of Home Care Program on Caregiver Burden with Stroke patients in Iran. While the evidence showed that stroke survivors are completely focused on physical recovery in the 1-month post discharge, the caregiver burden begins in the hospital and it increase through time [5, 8, 10]. Our results showed the same pattern in the control group. The first days after the discharge are crucial, because the family and the caregivers need to adopt with changes in the life of patients and their lifes. We started our intervention at the hospital and we continued it for up to 2 weeks to cover the psychological effects and lifestyle changes of the caregiver.

### Limitations

This was an experimental study that was conducted on eligible caregivers who had the mentioned inclusion criteria. The small sample size can reduce the generalization of the results. It is recommended to perform larger studies on a wider range of caregivers. Another limitation of this study was the significant difference in scores between the two groups before the intervention. ANCOVA is used to modify the effect of scores before the intervention, which showed that the supportive home care program was effective in the reduction of caregiver burden despite the higher scores in the control group before the intervention. However, it is recommended to use caregiver burden scores as a matching criterion in future studies.

## Conclusion

Stroke is a condition that has long-term effects on the patients and his/her caregivers. The family has a lot of problems coping with the new life that the disease imposes on them after the acute phase in the hospital. Caregiver Burden can increase after the discharge of stroke patients in the caregivers. The burden also can increase significantly on people who are caring for their patients at home. Providing support for home care providers along with proper and relative education can help to decrease or prevent the increase of caregiver burden. Future studies can help to further understand the methods that can help in the decrease of caregiver burden.

## Data Availability

All data will be available on request. All requests should send to the corresponding author email and they will be available within 1 week.

## Abbreviations

CB: Caregiver Burden
CBI: Caregiver Burden Inventory
CVA: Cerebrovascular Accident
ANCOVA: Analysis of Covariance
ADL: Activities of Daily Life
IS: Ischemic Stroke
HS: Hemorrhagic stroke
